# Using Routinely Collected Electronic Healthcare Record Data to Investigate Fibrotic Multimorbidity in England

**DOI:** 10.2147/CLEP.S463499

**Published:** 2024-06-24

**Authors:** Georgie M Massen, Hannah R Whittaker, Sarah Cook, Gisli Jenkins, Richard J Allen, Louise V Wain, Iain Stewart, Jennifer K Quint, On behalf of DEMISTIFI consortium, Andrew Thorley, Anna Duckworth, Ali-Reza Mohammadi-Nejad, Aloysious Aravinthan, Anthony Harbottle, Armando Mendez Villalon, Chris Scotton, Christopher Denton, Daniel Lea, Dorothee Auer, Ebrima Joof, Eleanor Cox, Elizabeth Eves, Elizabeth Robertson, Emma Blamont, Fasihul Khan, Georgie Massen, Gina Parcesepe, Gisli Jenkins, Gordon Moran, Guruprasad Aithal, Hilary Longhurst, Iain Stewart, Jane Paxton, Jennifer Quint, Karen Piper Hanley, Kate Frost, Leo Casmino, Lisa Chakrabarti, Louise Wain, Margot Roeth, Maria Kaisar, Martin Craig, Michael Nation, Mohammad Alireza Kisomi, Mujdat Zeybel, Neil Guha, Nicholas Selby, Nick Oliver, Nick Selby, Olivia C Leavy, Penny Gowland, Philip Quinlan, Rachel Chambers, Richard Allen, Richard Hubbard, Rob Slack, Rutger Ploeg, Sam Moss, Sara Fawaz, Scott Turner, Shauntelle Quammie, Simon Johnson, Stamatios N Sotiropoulos, Stuart Astbury, Susan Francis, Tom Giles, Valerie Quinn, Wendy Adams, Xin Chen, Zhendi Gong

**Affiliations:** 1 School of Public Health, Imperial College London, London, UK; 2 National Heart and Lung Institute, Imperial College London, London, UK; 3 Department of Population Health Sciences, University of Leicester, Leicester, UK; 4 NIHR Leicester Biomedical Research Centre, University of Leicester, Leicester, UK; 5 National Heart and Lung Institute, Imperial College London, London, United Kingdom; 6 University of Exeter, Exeter, United Kingdom; 7 Sir Peter Mansfield Imaging Centre, Mental Health and Clinical Neurosciences, School of Medicine, University of Nottingham, Nottingham NG7 2UH, United Kingdom; Institute for Health Research (NIHR) Nottingham Biomedical Research Ctr, Queens Medical Ctr, Nottingham, United Kingdom; 8 Nottingham Digestive Diseases Centre, Translational Medical Sciences, School of Medicine, University of Nottingham, Nottingham, UK; Nottingham University Hospitals NHS Trust and the University of Nottingham, Nottingham, UK; 9 Patient and Public Involvement and Engagement, Nottingham University Hospitals, Nottingham, United Kingdom; 10 Digital Research Service, University of Nottingham, Nottingham, United Kingdom; 11 Department of Clinical and Biomedical Sciences, University of Exeter,Exeter, United Kingdom; 12 Centre for Rheumatology, Royal Free Hospital and University College London, London, UK; 13 Digital Research Service, University of Nottingham, Nottingham, United Kingdom; 14 Mental Health & Clinical Neurosciences,School of Medicine, University ofNottingham, Nottingham, UK; Sir Peter Mansfield Imaging Centre,School of Medicine, University ofNottingham, Nottingham, UK; NIHR Nottingham Biomedical ResearchCentre, Queen’s Medical Centre,University of Nottingham, Nottingham,UK; 15 School of Life Sciences, University of Nottingham, Nottingham, United Kingdom, 2National Public Health Laboratories; Ministry of Health and Social Welfare, Banjul, The Gambia; 16 Sir Peter Mansfield Imaging Centre, School of Physics & Astronomy, University of Nottingham, Nottingham, UK; NIHR Nottingham BRC, Nottingham University Hospitals NHS Trust and the University of Nottingham, Nottingham, UK; 17 Diabetes UK, UK; 18 Diabetes UK, UK; 19 Scleroderma and Raynaud’s UK, UK; 20 Glenfield Hospital, University Hospitals of Leicester NHS Trust, Leicester, UK; 21 Imperial College London, London, United Kingdom; 22 Department of Population Health Sciences, University of Leicester, Leicester, UK; NIHR Leicester Biomedical Research Centre, Leicester, UK; 23 Gisli Jenkins, Margaret Turner Warwick Centre for Fibrosing Lung Disease, National Heart and Lung Institute, Imperial College London, United Kingdom; 24 Gordon W. Moran, NIHR Nottingham BRC, Nottingham University Hospitals NHS Trust and the University of Nottingham, Nottingham, UK; 25 Guruprasad P. Aithal, NIHR Nottingham BRC, Nottingham University Hospitals NHS Trust and the University of Nottingham, Nottingham, UK; 26 Dyskeratosis Congenita (DC) Action, UK; 27 National Heart and Lung Institute, Imperial College London, London, UK; 28 Dyskeratosis Congenita (DC) Action, UK; 29 National Heart and Lung Institute, Imperial College London, London, UK; 30 Division of Gastroenterology and Hepatology, Manchester University NHS Foundation Trust, Manchester, UK; 31 Patient and Public Involvement and Engagement, Nottingham University Hospitals, Nottingham, United Kingdom; 32 Sarcoidosis UK, UK; 33 School of Veterinary Medicine and Science, Sutton Bonington Campus, University of Nottingham, Nottingham, UK; Medical Research Council Versus Arthritis Centre for Musculoskeletal Ageing Research, Nottingham, UK; 34 Department of Population Health Sciences, University of Leicester, Leicester, UK; NIHR Leicester Biomedical Research Centre, University of Leicester, Leicester, UK; 35 University of Nottingham, Nottingham, UK; 36 Nuffield Department of Surgical Sciences, University of Oxford; 37 Sir Peter Mansfield Imaging Center, School of Medicine, University of Nottingham, Nottingham, UK ; Wellcome Centre for Integrative Neuroimaging, Nuffield Department of Clinical Neurosciences, University of Oxford, UK; Quantified Imaging, London, UK; 38 Kidney Research UK, UK; 39 Sir Peter Mansfield Imaging Centre, Mental Health and Clinical Neurosciences, School of Medicine, University of Nottingham, Nottingham NG7 2UH, United Kingdom; National Institute for Health Research (NIHR) Nottingham Biomedical Research Ctr, Queens Medical Ctr, Nottingham, United Kingdom; 40 NIHR Nottingham Biomedical Research Centre, Nottingham University Hospitals NHS Trust & University of Nottingham, Nottingham, UK; 41 NIHR Nottingham BRC, Nottingham University Hospitals NHS Trust and the University of Nottingham, Nottingham, UK; 42 Centre for Kidney Research and Innovation, University of Nottingham, Royal Derby Hospital Campus,Derby, UK; 43 Department of Medicine, Imperial College London, London, UK; 44 Centre for Kidney Research and Innovation, School of Medicine, University of Nottingham; 45 Department of Non-communicable Disease Epidemiology, The London School of Hygiene and Tropical Medicine, London, UK; Department of Health Sciences, University of Leicester,Leicester, UK; 46 Universiy of Nottingham, Sir Peter Mansfield Imaging Centre, Nottingham, United Kingdom; 47 The Digital Research Service, University of Nottingham, UK; 48 Centre for Inflammation and Tissue Repair, University College London, London, UK; 49 Department of Population Health Sciences, University of Leicester, Leicester, UK, 2NIHR Leicester Biomedical Research Centre, Leicester, UK; 50 University of Nottingham, Nottingham, UK; 51 Galecto, Stevenage, Hertfordshire, UK; 52 Nuffield Department of Surgical Sciences, University of Oxford, and Biomedical Research Centre Oxford; 53 Imperial College London, London, United Kingdom; 54 University of Nottingham, Nottingham, UK; 55 NIHR Nottingham BRC, Nottingham University Hospitals NHS Trust and the University of Nottingham, Nottingham, UK; 56 University of Norringham, Nottingham, United Kingdom; 57 Centre for Respiratory Research, NIHRRespiratory Biomedical Research Centre,School of Medicine, Biodiscovery Institute,University Park, University of Nottingham,Nottingham, UK; 58 Sir Peter Mansfield Imaging Centre, School of Medicine, University of Nottingham, Nottingham, UK; Wellcome Centre for Integrative Neuroimaging, University of Oxford, Oxford, UK; National Institute for Health Research (NIHR) Nottingham Biomedical Research Centre, Queens Medical Centre, Nottingham, UK; 59 Nottingham Digestive Diseases Centre, Translational Medical Sciences, School of Medicine, University of Nottingham, Nottingham, UK; NIHR Nottingham Biomedical Research Centre, Nottingham University Hospitals NHS Trust and the University of Nottingham, Nottingham, UK; 60 Sir Peter Mansfield Imaging Centre, School of Physics, University of Nottingham, UK; 61 The Digital Research Service & The Advanced Data Analysis Centre, University of Nottingham, UK; 62 Margaret Turner Warwick Centre for Fibrosing Lung Disease, National Heart and Lung Institute, Imperial College London, United Kingdom; 63 Action for Pulmonary Fibrosis, United Kingdom; 64 School of Computer Science, University of Nottingham, UK; 65 School of Computer Science, University of Nottingham, UK

**Keywords:** fibrosis, CPRD, electronic health records, multimorbidity, fibrotic multimorbidity, ONS

## Abstract

**Background:**

Electronic healthcare records (EHRs) are used to document diagnoses, symptoms, tests, and prescriptions. Though not primarily collected for research purposes, owing to the size of the data as well as the depth of information collected, they have been used extensively to conduct epidemiological research. The Clinical Practice Research Datalink (CPRD) is an EHR database containing representative data of the UK population with regard to age, sex, race, and social deprivation measures. Fibrotic conditions are characterised by excessive scarring, contributing towards organ dysfunction and eventual organ failure. Fibrosis is associated with ageing as well as many other factors, it is hypothesised that fibrotic conditions are caused by the same underlying pathological mechanism. We calculated the prevalence of fibrotic conditions (as defined in a previous Delphi survey of clinicians) as well as the prevalence of fibrotic multimorbidity (the proportion of people with multiple fibrotic conditions).

**Methods:**

We included a random sample of 993,370 UK adults, alive, and enrolled at a UK general practice, providing data to the CPRD Aurum database as of 1st of January 2015. Individuals had to be eligible for linkage to hospital episode statistics (HES) and ONS death registration. We calculated the point prevalence of fibrotic conditions and multi-morbid fibrosis on the 1st of January 2015. Using death records of those who died in 2015, we investigated the prevalence of fibrosis associated death. We explored the most commonly co-occurring fibrotic conditions and determined the settings in which diagnoses were commonly made (primary care, secondary care or after death).

**Results:**

The point prevalence of any fibrotic condition was 21.46%. In total, 6.00% of people had fibrotic multimorbidity. Of the people who died in 2015, 34.82% had a recording of a fibrotic condition listed on their death certificate.

**Conclusion:**

The key finding was that fibrotic multimorbidity affects approximately 1 in 16 people.

## Plain Language Summary

Fibrotic conditions are scarring conditions which impact the way an organ functions and eventually lead to organ failure. We studied routinely collected health data from GPs, hospitals, and death certificates to estimate the percentage of UK adults who had fibrotic diseases. We found that 1 in 5 people had at least one fibrotic disease, and we also found that 1 in 16 people had more than one fibrotic disease.

## Introduction

Fibrosis can affect any organ, and fibrotic conditions are characterised by excessive, uncontrolled deposition of extracellular matrix in the affected site, which in turn alters the tissue extracellular environment, leading to organ failure.^1^ There is growing evidence that high levels of inflammatory factors, such as IL14, are associated with an increased risk of idiopathic pulmonary fibrosis (IPF), with potential to target inflammatory factors to potentially prevent and treat fibrosis.^2^,^3^ Fibrosis is a defining feature of some conditions such as IPF and liver cirrhosis, however fibrosis can also develop in the latter stages of a condition such as diabetes.^4–6^ There is currently no cure for fibrotic diseases, and prognosis is poor and often comparable to end-stage cancer.^7^ It has been suggested that fibrotic conditions are associated with 45% of deaths in the industrialised world, therefore leading to significant healthcare burden; however, although this statistic is consistently quoted in fibrotic research, the source data for this estimate are unknown.^8^ As such, fibrotic diseases constitute a large research realm, with the possibility of shared mechanisms across multiple conditions.

Multimorbidity is defined as when a patient has two or more chronic health conditions.^9^ It is known that multimorbidity increases burden on healthcare systems as well as patients, therefore it is important to understand potential drivers of multimorbidity as well as phenotypes of patients most likely to be affected by multi-morbid conditions.^10^ We have adaptively termed “fibrotic multimorbidity” as the co-occurrence of 2 or more fibrotic conditions.

We previously conducted a Delphi study to gather clinical consensus on which diseases are fibrotic and identified 256 diseases which exhibit fibrotic manifestations.^11^ In this study, we defined and applied code lists for each of these diseases, to routinely collected electronic healthcare record data to determine the prevalence of fibrotic conditions as well as fibrotic multimorbidity.

The main objectives of this work were to calculate the prevalence of fibrotic conditions in the UK using the EHRs of a random sample of adults, and subsequently we looked to understand how many people in the sample suffered from multiple fibrotic conditions (fibrotic multimorbidity).

To assess the likelihood of co-occurring fibrotic multimorbidity, we focussed subsequent analysis on three single-organ fibrotic diseases of pulmonary fibrosis, liver cirrhosis, and urinary tract fibrosis as codes for these conditions would always indicate the presence of fibrosis as these conditions are characterised by fibrosis no matter the stage of progression.

We believe this to be the first piece of work investigating the co-occurrence of fibrotic conditions using large, detailed, routinely collected, representative healthcare data, highlighting the large prevalence of fibrotic conditions in the UK population.

## Methods

### Data Source

We used the Clinical Practice Research Datalink (CPRD) Aurum, a nationally representative database of anonymised primary care electronic healthcare records. CPRD Aurum contains the data of around 23% of the UK population and is representative of the UK population with regard to age, sex and deprivation.^12^ Clinical information is entered with SNOMED CT (systematised nomenclature of medicine-clinical terms) codes. Linked mortality data from the Office for National Statistics (ONS), and secondary care data from Hospital Episode Statistics (HES) were provided for this study by CPRD.

### Study Population

Beginning with a random sample of 1 million UK adults, we derived a random sample of 993,370 people who were eligible to be included in the analysis (the data of 6630 people was removed as they did not contribute at least 1 year of data). The patients had to be enrolled at the general practice before the 1st of January 2014 and still enrolled at the GP on the 1st of January 2015, they also had to be eligible for linkage to HES and ONS.

### Outcomes of Interest

Fibrotic conditions were previously defined in a Delphi study consisting of three survey rounds using clinical consensus as an indicator of whether a condition was fibrotic.^2^ The following organ/disease groups were used: pulmonary fibrosis, urinary tract fibrosis, liver cirrhosis, diabetes, blood vessel fibrosis, atherosclerosis, bile duct fibrosis, cardiomyopathy, integumentary fibrosis, intestinal/pancreatic fibrosis, lymphatic fibrosis, peritoneal fibrosis, reproductive fibrosis, skeletal fibrosis, systemic fibrosis, and fibrosis of the cardiac valves. SNOMED CT codes were used to identify these conditions in primary care data whilst ICD 10 codes were used to identify diagnoses from both HES and ONS data sources. These codelists are available on Github: https://github.com/NHLI-Respiratory-Epi/Fibrotic-multimorbidity

### Statistical Methods

We initially described the baseline characteristics (age and sex), of each of the subpopulations diagnosed with fibrotic conditions and reported the point prevalence of these conditions as well as the point prevalence of multi-morbid fibrosis as of the 1st of January 2015. We also calculated the proportion of deaths in 2015 which had a record of a fibrotic condition within one of the positions of cause of death (ONS records can contain up to 15 codes which can be used to detail conditions which contributed towards cause of death).

Using the data of people who died in 2015, we investigated the location that diagnoses were made. We defined the location as either a primary care setting (if the earliest date was found in the CPRD observation file), a secondary care setting (if the earliest date was found in a HES admitted patient care file), or post-mortem (if the diagnosis was only found in one of the codes for any of the positions in the cause of death). The codes present in ONS death records are in the form of ICD 10 codes, the same coding system used by hospitals. Therefore, we applied the same codelists to the ONS records that we used in the HES records.

We then focused on three subpopulations: people with a diagnosis of pulmonary fibrosis, those with urinary tract fibrosis and those with liver cirrhosis. These conditions were selected as they were deemed to always be fibrotic and were therefore best poised to be included in analysis of fibrotic multimorbidity as codes for these conditions would always identify the presence of fibrosis. Confidence intervals of 99% were applied to support robust associations. Logistic regression was performed adjusting for age (quartiles) and sex to investigate the relationship between one of the three selected fibrotic conditions of interest (exposures) and the other 13 fibrotic conditions (outcomes). We explored the five most commonly occurring fibrotic conditions, both prior and post diagnosis of each of these conditions, and reported the median time between these. Finally, we calculated the odds of being diagnosed with each of the fibrotic conditions, given a diagnosis of one of pulmonary fibrosis, urinary tract fibrosis, or liver cirrhosis, respectively, for example, the odds of being diagnosed with reproductive fibrosis given a diagnosis of pulmonary fibrosis.

All analyses were performed using Stata version 17. If the prevalence of a fibrotic condition in the random sample of 1 million was less than 0.05%, it was removed from the analysis to preserve anonymity.

## Results

Of the 993,370 people included in this study, the median age was 46 years and 50.26% of the population were female. The median follow-up time for all patients was 10 years (3963 days, IQR: 1875–8268). Cardiac cirrhosis, cardiac fibrosis, fibrosis of the spleen, fibrosis of the nervous system, appendix fibrosis, and oral fibrosis were not included in the analysis as the prevalence of these conditions was less than 0.05%.

The overall point prevalence of fibrotic diseases in the entire sample was 21.46% (99% CI: 21.35–21.56). As of the 1st of January 2015, the median age of people with prevalent fibrotic disease was 65 years (IQR: 52–77) and 53.32% of those with at least one fibrotic condition were female (Supplementary Table 1). In total, 15.46% of people had a record of one fibrotic disease whilst 6.00% of people had two or more fibrotic conditions in their records and therefore had fibrotic multimorbidity (Table 1).

**Table 1:** Number of Fibrotic Conditions

No. Conditions	Frequency	Percentage	Fibrotic Multimorbidity
0	780,201	78.54	
1	153,527	15.46	
2	41,473	4.17	
3	12,844	1.29	
4	3,934	0.4	6.00%
5	1,061	0.11	
6	250	0.03	
7+	80	<0.01	
Total	993,370	100	

**Notes**: The number of fibrotic conditions experienced by the people in the cohort, using CPRD and HES Admitted Patient Care data.

Of those within the sample who died in 2015 (n = 21,857), 34.82% had a record of at least one fibrotic condition in at least one of the positions of cause of death, whilst 7.85% had two fibrotic conditions present on their death certificate. Of the people who died in 2015, 6.89% had a fibrotic condition recorded as their primary cause of death. Of 7611 people who had a fibrotic condition listed on their death record, 19.42% (n = 1478) did not have a record of a fibrotic condition in their GP or hospital records.

**Figure 1:**
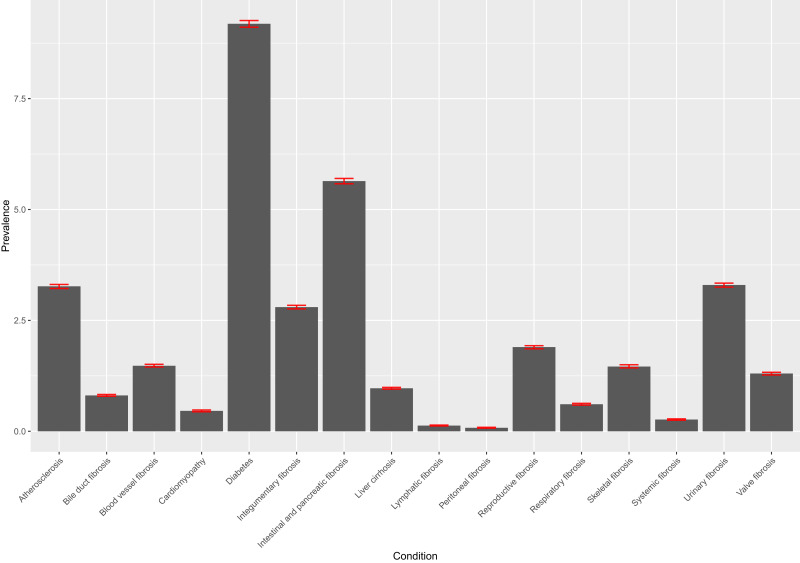
Barplot of prevalence of fibrotic conditions. The prevalence of fibrotic conditions derived from both CPRD and HES admitted patient care, displayed alongside a 99% confidence interval.

Diabetes was the most prevalent fibrotic condition (9.19%, 99% CI: 9.11–9.26), followed by fibrosis of the intestines/pancreas (5.64%, 99% CI: 5.58–5.70) (Figure 1). When investigating the prevalence of multi-morbid fibrosis, 3 disease combinations were found to have a prevalence greater than 1% (urinary tract fibrosis and diabetes, diabetes and atherosclerosis, diabetes and intestinal/pancreatic fibrosis). Also, three disease combinations had a prevalence lower than 1% but greater than 0.5%: urinary tract fibrosis and atherosclerosis, urinary tract fibrosis and intestinal/pancreatic fibrosis, atherosclerosis and intestinal/pancreatic fibrosis (Supplementary Table 2).

**Figure 2:**
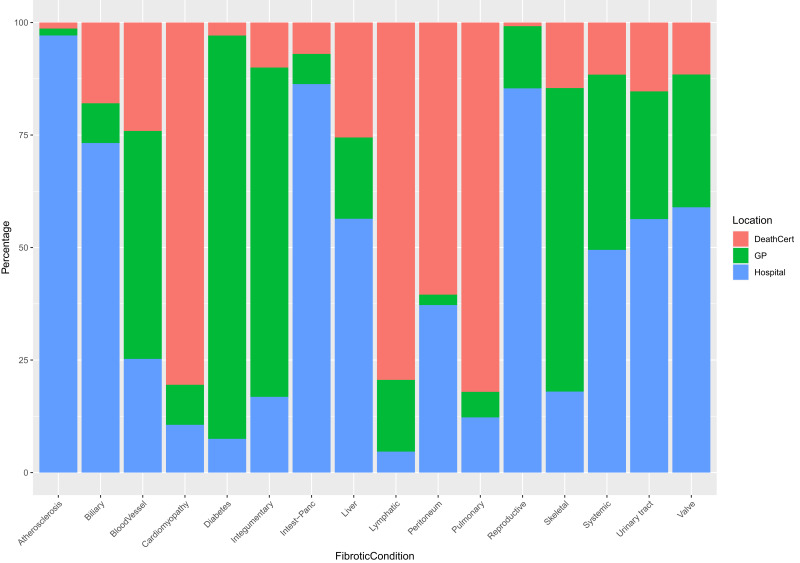
Location of diagnoses. Using the earliest entry date of diagnoses of fibrotic conditions, the proportions of diagnoses being made in primary and secondary care settings as well as on death certificate was calculated. This analysis used the data of people who died in 2015.

Most fibrotic conditions (72.83%) were diagnosed in either primary or secondary care settings (Figure 2 and Supplementary Table 3). However, of the people with a record of cardiomyopathy 80.50% of their diagnoses of cardiomyopathy were first reported on their death certificate, similarly of the people with pulmonary fibrosis, 82.06% of the diagnoses were first reported on death certification; of the 301 people who died in 2015 and were diagnosed with lymphatic fibrosis, 79.40% of the diagnoses were first recorded on death certificates. Of the diabetes diagnoses, 89.62% were diagnosed in a primary care setting whilst 65.11% of skeletal fibrosis diagnoses were also made in primary care. Most diagnoses of atherosclerosis, biliary fibrosis, and fibrosis of the intestines/pancreas were made in secondary care (97.11%, 73.22% and 86.31%, respectively).

**Figure 3:**
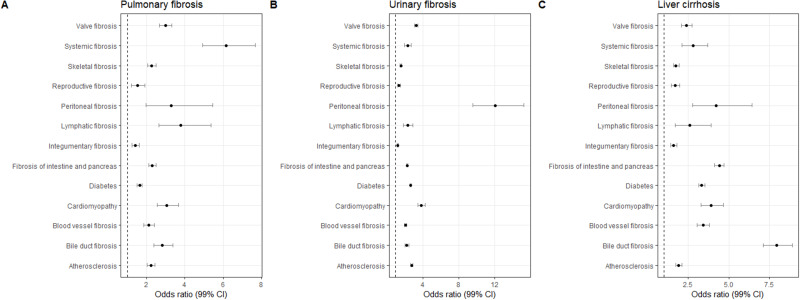
Forest plot of odds ratio of being diagnosed with a fibrotic condition. Using logistic regression, the log-odds ratio of having a record of a fibrotic condition, given a record of: (**A**) pulmonary fibrosis, (**B**) urinary tract fibrosis, (**C**) liver cirrhosis.

There were significantly greater odds of being diagnosed with one of systemic fibrosis, lymphatic fibrosis and peritoneal fibrosis when comparing people with and without a diagnosis of pulmonary fibrosis (Unadjusted analyses: OR systemic: 9.66, 99% CI: 7.55–12.36, OR lymphatic: 8.07, 99% CI: 5.55–11.73, OR peritoneal: 3.68, 99% CI: 1.79–7.58), (Adjusted analyses: ORadj: 6.16, 99% CI: 4.94–7.69, ORadj: 3.78, 99% CI: 2.65–5.38 and ORadj: 3.28, 99% CI: 3.28, 99% CI: 1.98–5.46, respectively) (Figure 3). A significantly greater odds of being diagnosed with peritoneal fibrosis was observed when comparing people diagnosed with urinary tract fibrosis compared with those not diagnosed with urinary tract fibrosis (OR: 15.32, 99% CI: 12.32–19.06; ORadj: 12.04, 99% CI: 9.53–15.21). Significantly greater odds of being diagnosed with bile duct fibrosis were identified when comparing people diagnosed with liver cirrhosis with people who did not have a diagnosis of liver cirrhosis in their records (OR: 9.41, 99% CI: 8.31–10.66; ORadj: 7.95, 99% CI: 7.10–8.90).

A total of 1741 (28.74%) people diagnosed with pulmonary fibrosis and another fibrotic condition and were included in analysis of the 5 most commonly co-occurring fibrotic conditions, both prior and post diagnosis (Supplementary Figure 1). Post-diagnosis, the most common condition was fibrosis of the intestines/pancreas which 101 people were diagnosed with, the median time between these diagnoses was 2.1 years (IQR: 0.3–5.2). The most common diagnosis prior to pulmonary fibrosis was skeletal fibrosis (n = 397) the median time between these diagnoses was 7.5 years (IQR: 2.3–15.9).

A total of 9827 (29.99%) people had a record of urinary tract fibrosis and another fibrotic condition (Supplementary Figure 2). The most commonly occurring fibrotic condition after diagnosis of urinary tract fibrosis was intestinal/pancreatic fibrosis (n = 815) for which the median time between diagnoses was 1.5 years (IQR: 0.0–3.9). The most diagnosed fibrotic condition prior to urinary tract fibrosis was diabetes (n = 3641), the median time between diagnoses was 8.1 years (IQR: 3.3–14.4).

Of the 2604 (26.95%) people who had a diagnosis of liver cirrhosis and another fibrotic condition in their records, diabetes was the most diagnosed fibrotic condition both before and after diagnosis of liver cirrhosis (Supplementary Figure 3). In total 353 people were diagnosed with diabetes after being diagnosed with liver cirrhosis, the median time between these diagnoses was 0.6 years (IQR: 0.0–4.0). A total of 667 people were diagnosed with diabetes prior to being diagnosed with liver cirrhosis. The median time between these diagnoses was 4.4 years (IQR: –1.6–9.0).

## Discussion

Using routinely collected electronic healthcare records, we have determined the prevalence of fibrotic conditions in a random sample of adults in England, as of 2015. Of the 993,370 people included in this analysis, 21.46% had at least one diagnosis of a fibrotic condition, whilst 6.00% of the cohort had two or more diagnoses of fibrotic conditions and therefore fibrotic multimorbidity. The most prevalent fibrotic condition was found to be diabetes. Investigation of the setting in which conditions were first diagnosed identified that diabetes diagnoses were commonly made in primary care as well as diagnoses of skeletal fibrosis, conversely diagnoses of atherosclerosis and intestinal/pancreatic fibrosis were made in secondary care. We found that of the people in the sample that died in 2015, 34.82% had a recording of a fibrotic condition listed on their death certificate, whilst 6.89% of deaths were due to a fibrotic condition (as recorded in the primary cause of death). The United States Government estimated that around 45% of deaths in the USA were ‘attributed to fibrotic disorders’;^8^ we have shown that of a random sample of deaths in England in 2015, 34.82% of the records of these people included a code for a fibrotic condition in at least one of the positions of cause of death.

We demonstrated that the odds of being diagnosed with an additional fibrotic condition were greater for people with a fibrotic condition compared with those without, using three example conditions (of pulmonary fibrosis, urinary tract fibrosis, or liver cirrhosis).

The prevalence of fibrotic conditions derived from this random sample of adults is similar to previously recorded prevalence values; for instance, we identified the prevalence of diabetes in our cohort as 9.19%, whilst Diabetes UK reported the prevalence in the UK as of 2015 being 9%.^13^ This demonstrates that the result of our analysis is likely to be representative of the prevalence of fibrotic conditions in England. However, in some instances, it is harder to directly compare the derived prevalence as the groups were mostly defined based on the affected organ; intestinal/pancreatic fibrosis was found to be prevalent in 5.64% of the sample and this broad definition including conditions such as Crohn’s diseases, colitis, inflammatory bowel disease and pancreatitis. Crohn’s and colitis are estimated to effect 0.81% of the population.^14^ The prevalence of irritable bowel disease in the UK has been previously found to be 1.42% in 2016 whilst the prevalence of pancreatitis (determined using UK Biobank) was found to be 0.163%.^15^ Integumentary fibrosis was found in our sample to be prevalent in 2.80%; previous research has found that Raynaud’s phenomenon (included diseases in our definition of integumentary fibrosis) affects around 5% of the population.^16^ It has been previously found that lung damage and microvascular complications are associated with systemic sclerosis.^17^ One in ten women in the UK of reproductive age suffers from endometriosis,^18^ and reproductive fibrosis which included endometriosis was found to be recorded in 1.90% of the random sample of UK adults.

Many of the conditions identified in the previous Delphi survey are not idiopathic and instead are a result of chronic organ inflammation.^9^ This adds complexity when trying to understand the co-occurrence of these conditions, as it may not be due to an underlying “fibrotic” mechanism but instead randomness, other ageing processes or other causes which are yet to be identified. As we used routinely collected electronic healthcare records, we were unable to study genetic markers or biomarkers. As we used electronic healthcare records, we conducted a Delphi survey of clinicians as these are the professionals who enter the data into the records. We have not studied this from a pathological angle, a condition that a clinician may deem to be fibrotic and could be contested by a pathologist.

### Non-Clinical Implications

We have shown that 1 in 5 adults in the UK have fibrotic conditions, this is a significant statistic which highlights the burden of fibrotic conditions on both patients and healthcare systems. As a result, it is important that resources be sufficiently allocated for the treatment and management of these patients in both primary and secondary care services. Also, we found that 1 in 16 people had multiple fibrotic conditions. This indicates that there could be overlapping and potentially contradicting care plans in place and risking polypharmacy, and therefore, we encourage care providers to conduct multidisciplinary team meetings between specialities to provide optimal care.

### Clinical Implications

We have shown that many of the fibrotic conditions were first reported on death certification rather than in primary or secondary care records, and it is highly likely that the conditions are not being diagnosed and are therefore untreated. As we have shown greater odds in being diagnosed with other fibrotic conditions in people with a diagnosis of either pulmonary fibrosis, urinary tract fibrosis, and liver cirrhosis, it is important to understand what factors increase the risk of developing multiple fibrotic conditions such as genetics or medications. As such, it is important to consider other fibrotic condition diagnoses and investigations in patients with one of these three conditions.

## Strengths and Limitations

CPRD Aurum data is known to be representative of the UK population with respect to age, sex, ethnicity, and social deprivation. This database is derived from entries that GPs create, however free text is not available, we triangulated this data by linking the data with hospitalisations and death records (where possible) to gain the most detailed possible account of people’s health. The prevalence estimates provided here were derived from a random sample of 993,370 adults and are therefore estimates of the prevalence of these diseases within the British population. As some of the conditions were first reported on the death certificate, we have likely underestimated the prevalence of conditions on the 1st of January 2015 as it is possible that these underlying undiagnosed conditions would have been found in these people at that point in time if investigations had occurred. We have used broad definitions of fibrotic disease, sub-grouped based on the affected organs, therefore these data cannot be interpreted for individual conditions, however, we have been able to descriptively analyse the data, which is available using broad overarching methodologies. The fibrotic conditions which were investigated were defined by clinicians, however some of these conditions may only be fibrotic in the later stages of the disease, however it was not possible to define the stages/severity of disease in this analysis. When applying logistic regression models, both age and sex were adjusted for, however we acknowledge that fibrotic conditions could have a multitude of comorbidities as well as other contributing factors including and not limited to smoking status and alcohol consumption which could confound the result. However, as we were analysis groups of fibrotic conditions, it was not possible to account for all possible confounders.

## Conclusion

Using a large sample of UK adults, we have investigated the prevalence of fibrotic conditions in electronic healthcare records as well as the prevalence of fibrotic multimorbidity. We found that as of 2015, the prevalence of fibrotic conditions in a random sample of UK adults was 21.46%, and the prevalence of fibrotic multimorbidity was 6.00%. We have analysed information of those within the cohort who died in 2015, of which 34.82% had at least one fibrotic condition recorded on their death certification. Using three cohorts we showed that there is an increased chance of being diagnosed with a fibrotic condition if you already have a fibrotic condition. We believe this to be the first piece of work investigating the co-occurrence of fibrotic conditions using large, routinely collected, representative healthcare data, highlighting the large prevalence of fibrotic conditions in the UK population as well as the co-occurrence of fibrotic conditions. It is important that fibrotic conditions be diagnosed at the earliest opportunity, with diagnoses being made in life so that interventions such as treatment or palliation can be accessed at the earliest point.

## Data Availability

Data may be obtained from a third party and are not publicly available. Linked pseudonymised mortality data from the Office for National Statistics (ONS) and secondary care data from Hospital Episode Statistics (HES) were provided for this study by CPRD for patients in England. Data are linked by NHS Digital, the statutory trusted third party for linking data, using identifiable data held only by NHS Digital. Select general practices consent to this process at a practice level, with individual patients having the right to opt-out. Use of HES and ONS data is Copyright © (2018), reused with the permission of The Health & Social Care Information Centre, all rights reserved. Data are available on request from the CPRD. Their provision requires the purchase of a license, and this license does not permit the authors to make them publicly available to all. This work used data from the version collected in May 2022 and has clearly specified the data selected in each Methods section. To allow identical data to be obtained by others, via the purchase of a license, the code lists will be provided upon request. Licenses are available from the CPRD (http://www.cprd.com): The Clinical Practice Research Datalink Group, The Medicines and Healthcare products Regulatory Agency, 10 South Colonnade, Canary Wharf, London E14 4PU.

## References

[1] Rittié L. Fibrosis: methods and Protocols. In: *Methods in Molecular Biology*. New York: Springer New York.; 2017.

[2] Jia Q, Lei Y, Chen S, Liu S, Wang T, Cheng Y. Circulating inflammatory cytokines and risk of idiopathic pulmonary fibrosis: a Mendelian randomization study. *BMC Pulm Med*. 2023;23(1):369. doi:10.1186/s12890-023-02658-3 37789433 PMC10548733

[3] Salton F, Ruaro B, Confalonieri P, Confalonieri M. Epithelial-mesenchymal transition: a major pathogenic driver in idiopathic pulmonary fibrosis? *Medicina*. 2020;56(11):608. doi:10.3390/medicina56110608 33202716 PMC7697350

[4] Barratt SL, Creamer A, Hayton C, Chaudhuri N Idiopathic Pulmonary Fibrosis (IPF): an Overview; 2018. Available from: https://www.mdpi.com/2077-0383/7/8/201. Acce 10.3390/jcm7080201 PMC6111543 30082599

[5] Bataller R, Brenner DA. Liver fibrosis. *J Clin Invest*. 2005;115(2):209–218. doi:10.1172/JCI24282 15690074 PMC546435

[6] Tuleta I, Frangogiannis NG. Diabetic fibrosis. *Bioch et Bioph Acta*. 2021;1867(4):166044. 10.1016/j.bbadis.2020.166044 PMC7867637 33378699

[7] Wernig G, Chen SY, Cui L, et al. Unifying mechanism for different fibrotic diseases. *Proc Natl Acad Sci*. 2017;114(18):4757–4762. doi:10.1073/pnas.1621375114 28424250 PMC5422830

[8] Wynn TA. Fibrotic disease and the T(H)1/T(H)2 paradigm. *Nat Rev Immunol*. 2004;4(8):583–594. doi:10.1038/nri1412 15286725 PMC2702150

[9] Navickas R, Petric VK, Feigl AB, Seychell M. Multimorbidity: what do we know? What should we do? *J Comorb*. 2016;6(1):4–11. doi:10.15256/joc.2016.6.72 29090166 PMC5556462

[10] Starfield B. Challenges to primary care from co- and multi-morbidity. *Prim Health Care Res Develop*. 2011;12(1):1–2. doi:10.1017/S1463423610000484 21426609

[11] Massen GM, Allen RJ, Leavy OC, et al. Classifying the unclassifiable—a Delphi study to reach consensus on the fibrotic nature of diseases. *QJM: Int J Med*. 2023;116(6):429–435. doi:10.1093/qjmed/hcad050 PMC10250078 37004203

[12] Wolf A, Dedman D, Campbell J, et al. Data resource profile: clinical practice research datalink (CPRD) Aurum. *Int J Epidemiol*. 2019;48(6):1740–1740g. doi:10.1093/ije/dyz034 30859197 PMC6929522

[13] Diabetes UK. New diabetes prevalence figures for England; 2023. Available from: https://www.diabetes.org.uk/about_us/news/new-diabetes-prevalence-figures-for-england. Accessed May 29, 2024.

[14] Chron’s & Colitis UK. New research shows over 1 in 123 people in UK living with Crohn’s or Colitis; 2023. Available from: https://crohnsandcolitis.org.uk/news-stories/news-items/new-research-shows-over-1-in-123-people-in-uk-living-with-crohn-s-or-colitis. Accessed May 29, 2024.

[15] Freeman K, Ryan R, Parsons N, Taylor-Phillips S, Willis BH, Clarke A. The incidence and prevalence of inflammatory bowel disease in UK primary care: a retrospective cohort study of the IQVIA medical research database. *BMC Gastroenterol*. 2021;21(1):139. doi:10.1186/s12876-021-01716-6 33771127 PMC8004426

[16] Haque A, Hughes M. Raynaud’s phenomenon. *Clin Med Lond*. 2020;20(6):580–587. doi:10.7861/clinmed.2020-0754 33199324 PMC7687329

[17] Ruaro B, Confalonieri M, Salton F, et al. The relationship between pulmonary damage and peripheral vascular manifestations in systemic sclerosis patients. *Pharmaceuticals*. 2021;14(5):403. doi:10.3390/ph14050403 33922710 PMC8145021

[18] Rogers PAW, D’Hooghe TM, Fazleabas A, et al. Priorities for endometriosis research: recommendations from an international consensus workshop. *Reprod Sci*. 2009;16(4):335–346. doi:10.1177/1933719108330568 19196878 PMC3682634

